# Characterization of *Riemerella anatipestifer* Strains Isolated from Various Poultry Species in Poland

**DOI:** 10.3390/antibiotics12121648

**Published:** 2023-11-22

**Authors:** Anna Nowaczek, Marta Dec, Dagmara Stępień-Pyśniak, Jarosław Wilczyński, Renata Urban-Chmiel

**Affiliations:** 1 Department of Veterinary Prevention and Avian Diseases, Faculty of Veterinary Medicine, University of Life Sciences in Lublin, 20-033 Lublin, Poland; marta.dec@up.lublin.pl (M.D.); dagmara.stepien@up.lublin.pl (D.S.-P.); renata.urban@up.lublin.pl (R.U.-C.); 2 Veterinary Diagnostic Laboratory Lab—Vet, 62-080 Tarnowo Podgórne, Poland; wilczynski@lab-vet.com.pl

**Keywords:** *Riemerella anatipestifer*, MALDI-TOF MS, phylogeny, MIC, poultry, 16S RNA, *rpo*B, antibiotic resistance

## Abstract

*Riemerella anatipestifer* (*R. anatipestifer*) is one of the common pathogens found in poultry flocks, resulting in serious economic losses for the poultry industry due to high mortality, reduced growth rate, poor feed conversion, increased condemnations, and high treatment costs. The aim of this study was to phenotypically characterize phylogenetic relationships and assess the presence of resistance gene strains of *R. anatipestifer* obtained from various poultry species in Poland. A total of 57 isolates of *Riemerella* were included in this study. A polymerase chain reaction (PCR) and matrix assisted laser desorption ionization mass spectrometry (MALDI-TOF MS) were used for identification of the strains. The phylogenetic relationship of the *R. anatipestifer* isolates was determined by analysing the *rpo*B gene sequence. The susceptibility to antibiotics was evaluated by minimum inhibitory concentration (MIC) in liquid media. All of the field strains of *R. anatipestifer* were grouped into one of two clades resulting from *rpo*B gene sequencing. High MIC_50_ and MIC_90_ values were obtained for gentamycin, amikacin, and colistin. Low MIC_50_ and MIC_90_ values were obtained for amoxicillin cefuroxime, cefoperazone, piperacillin, and trimethoprim/sulfamethoxazole. Among the resistance genes, *tet(X)* and *ermF* were identified most frequently. This is the first phenotypic characterization of *R. anatipestifer* strains obtained from poultry flocks in Poland.

## 1. Introduction

*Riemerella anatipestifer* (*R. anatipestifer*) is a Gram-negative, non-sporulating rod occurring singly, in pairs, and occasionally in chains, without cilia, bipolar staining bacterium which is responsible for infections in birds involving fibrinous exudative serositis and particularly affects the pericardial sac, air sacs, and liver [1,2]. The cells vary from 0.2 to 0.4 mm in width and 1 to 5 mm in length [2]. Colonies on blood agar are 1–2 mm in diameter, greyish, convex, dew drop-like, and smooth. Bacteria do not grow on MacConkey agar, and little or no growth on Litmus Lactose agar has been observed [3]. The phylogenetic and taxonomic position of this bacterium was proposed by Segers et al. [4], who suggested that it should be classified as a separate genus within the family *Flavobacteriaceae*. *R. anatipestifer*, together with *Riemerella columbina* (*R. columbina*) and *Riemerella columbipharyngis* (*R. columbipharyngis*), currently form the separate genus *Riemerella*, belonging to the family *Weeksellaceae* [5,6]. Septicaemia anserum exsudativa, caused by *R. anatipestifer*, is a disease common in many Asian countries with intensive rearing of ducks and geese [7,8,9]. The infection affects young birds, especially those at 1 to 8 weeks of age [2]. In addition to waterfowl, other species of birds can be susceptible to infection, including Galliformes, with the highest susceptibility observed in turkeys [10]. Chickens, quails, and pheasants are less frequently affected by the illness [11]. *R. anatipestifer* infection causes high economic losses due to high mortality, poor growth, increased feed consumption, and high treatment costs [12]. Most strains of *R. anatipestifer* are susceptible to amoxicillin, enrofloxacin, chloramphenicol, and ceftiofur. More than 90% of the strains are resistant to aminoglicosides and antibiotics from the polymyxin group [2,11]. It can be observed that the number of manuscripts describing *R. anatipestifer* infections in European countries has increased in the last two decades [1,10,13,14,15,16]. The number of poultry farms in Poland has been gradually increasing in recent decades. Although Poland is the largest producer of poultry meat from the region of Central and Eastern Europe [17], there are not enough data about *R. anatipestifer* strains isolated from poultry in the country. Given the potential threat of the spread of *Riemerella* bacteria among poultry and the increasingly observed phenomenon of bacterial resistance to antibiotics, it is particularly necessary to know the sensitivity of strains found in this area. The aim of this study was to isolate strains of *R. anatipestifer* from various poultry species in Poland, characterize their biochemical features, assess their sensitivity to drugs and the presence of resistance genes, and also determine the phylogenetic relationship.

## 2. Results

### 2.1. Identification of R. anatipestifer Isolates

Based on the morphological characteristics of the colonies grown on Columbia agar, we obtained fourteen isolates from turkeys, seven from ducks, seven from geese, and two from chickens (n = 30). Additionally, the study included 27 strains of which the species of bird from which they were isolated is unknown.

Identification as *R. anatipestifer* was confirmed by MALDI-TOF MSat the species or genus level for all isolates. The probability of correct identification in the MALDI Biotyper 3.1 system is expressed in points. For 52 bacterial isolates, the identification score was higher than 2.000. A score above 2.300 was obtained for 27 isolates, which indicates highly probable identification at the species level. Highly probable identification at the genus level and probable identification at the species level (2.000–2.299) was obtained for 25 isolates. For five isolates which were morphologically similar to *R. anatipestifer*, the score was below 1.999, so they could only be identified at a genus level by mass spectrometry (Table S1). Analysis of the electrophoretic profiles of the PCR products (16S rRNA and species-specific primers for RA L-17 and RA R-354) confirmed the presence of reaction products of 1460 bp and 338 bp in all isolates (n = 57).

### 2.2. Phylogenetic Relationship of the R. anatipestifer Isolates

**Figure 1:**
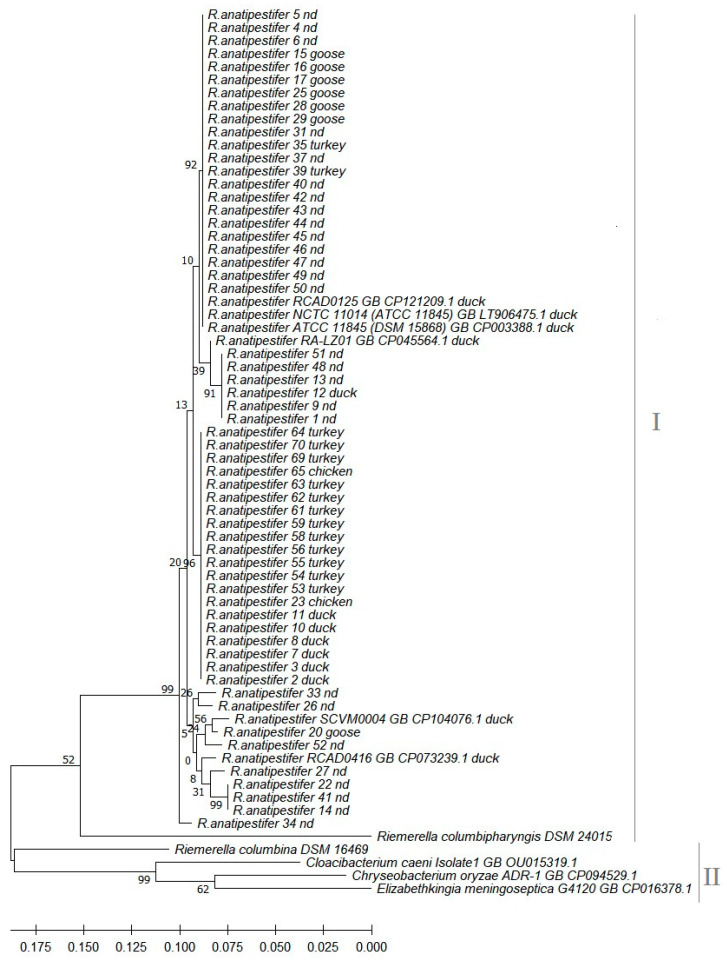
Phylogenetic tree based on the *rpo*B gene sequences of 57 wild-type avian *Riemerella* sp. strains built by the maximum likelihood method (MEGA X bioinformatic tool). The percentage of replicate trees in which the associated taxa were clustered together in the bootstrap test (500 replicates) is shown next to the branches. Scale bars show genetic distance. nd—no data.

The phylogenetic relationship of the *R. anatipestifer* isolates (n = 57) determined by comparative analysis of the *rpo*B gene sequence is presented in Figure 1. Apart from field strains, reference strains of *R. anatipestifer* (n = 6), *R. columbina* (n = 1), *R. columbipharyngis* (n = 1), and other species closely related to *R. anatipestifer* belonging to the family *Weeksellaceae* (n = 3) were used in the analysis. The strains formed two main clades, the first of which included all strains of *R. anatipestifer* and the *R. columbipharyngis* reference strain (I), while the other contained the *R. columbina* reference strain and the three reference strains of other species closely related to *R. anatipestifer*, belonging to the family *Weeksellaceae* (II). Comparative analysis of the *rpo*B gene sequences of the 57 strains revealed the occurrence of 40 variable sites in a segment of 657 nucleotides. The clade grouping of the *R. anatipestifer* strains had sixteen nodes, two of which were especially numerous—one with twenty-two field strains, including six from geese, and three reference strains (RCAD0125 GB, NCTC 11014, and ATCC 11845), and the other with twenty field strains, isolated mainly from turkeys and ducks.

### 2.3. Biochemical Profiles of R. anatipestifer Isolates

A biochemical analysis of the isolates resulted in three different reaction profiles. In the vast majority of cases the dominant API codes were 20NE 0010004 (n = 33) and 0210004 (n = 21). An API code 20NE 0000004 was characteristic of three strains. All isolates (n = 57) showed a positive reaction for oxidase and catalase. Most isolates (n = 51) had a positive reaction for the proteolytic enzyme gelatinase. More than one third of isolates (n = 21) were positive for urease.

### 2.4. Antibiotic Resistance Profiles of R. anatipestifer Isolates

Analysis of the MIC_50_ and MIC_90_ values of 14 antibiotics for the *R. anatipestifer* isolates showed significant variation in their susceptibility. High MIC_50_ and MIC_90_ values of ≥128 μg/mL were obtained for gentamicin, amikacin, and colistin. For two chemotherapeutics, erythromycin and enrofloxacin, the MIC_90_ values were fairly high, at 32 and 16 μg/mL, respectively, while the MIC_50_ was low, at 0.5 μg/mL, for both chemotherapeutics. For chloramphenicol and ciprofloxacin, the MIC_90_ values were 8 μg/mL and MIC_50_ = 2 μg/mL and 1 μg/mL, respectively. For tetracycline, the MIC_90_ was 4 μg/mL and the MIC_50_ was 1 μg/mL. The MIC_90_ and MIC_50_ values for amoxicillin were 2 μg/mL and 0.5 μg/mL, respectively. Low MIC_50_ = 0.125 μg/mL and MIC_90_ = 0.5 μg/mL values were obtained for cefoperazone and piperacillin. For cefuroxime, the MIC_50_ and MIC_90_ were 0.063 μg/mL and 0.125 μg/mL, respectively. The mixture of trimethoprim and sulfamethoxazole had an MIC_90_ value of 1/19 μg/mL and an MIC_50_ of 0.125/2.38 μg/mL (Table 1). More detailed MIC results for the individual bacteria can be found in the Supplementary Materials (Table S2).

### 2.5. Resistance Genes on R. anatipestifer Isolates

The distribution of resistance genes is shown in Table 2. The presence of at least one resistance gene used in the study was detected for 72% of the *R. anatipestifer* isolates. Among the aminoglycoside resistance genes, *aph(3′)-VII*, *aac(3′)-IV*, *aadA*, and *strA/strB* were identified. The *aph(3′)-VII* gene that causes resistance to amikacin, neomycin, and kanamycin was detected in seven isolates. The *aac(3′)-IV* gene that mediates resistance to gentamicin, neomycin, and tobramycin was detected in two isolates. Regarding streptomycin resistance genes, which belong to the aminoglycoside group, *aadA*, *strA,* and *strB,* were found in two of the examination isolates. Among the tetracycline resistance genes, *tet(A)*, *tet(B)*, and *tet(X)* were detected. The *tet(X)* gene occurred in forty isolates, and the *tet(A)* and *tet(B)* genes were detected in six and three isolates, respectively. The *ermF* gene was found in 14 isolates. Among the chloramphenicol and florfenicol resistance genes, the *cmlA* gene was detected in four isolates. For the four β-lactamase resistance genes used in the study, the *bla_TEM_* gene was confirmed in two strains. The *sulI* gene was found in one isolate. None of the *R. anatipestifer* isolates contained the *aac(6′)-Ib*, *aac(3′)-IIc*, *bla_OXA_*, *bla_CTX-M_*, *bla_SHV_*, *cat2*, *flor*, *sulII*, *sulIII*, and *dhfr1* genes (Table 2).

## 3. Discussion

Techniques based on molecular biology are a commonly used diagnostic tool for the identification of microorganisms. They include polymerase chain reaction (PCR) and gene sequencing, which have largely replaced techniques involving the identification of microorganisms using phenotypic methods. The technique of identifying microorganisms based on their protein profile by MALDI-TOF MS is increasingly used for various bacterial isolates, including *Campylobacter*, *Lactobacillus,* and *Enterococcus* [18,19,20], as well as in the case of non-fermenting bacteria, including those of the family *Weeksellaceae*, which includes the genus *Riemerella* [21,22]. Mass spectrometry can also be successfully used to identify strains of *R. anatipestifer*, and the sensitivity of this method is reflected by the results of the present study, in which we obtained a high identification score in MALDI-TOF MS for nearly all bacterial isolates. This has been confirmed by other authors as well [1,23,24]. In addition, protein profile analysis by mass spectrometry enables interspecific differentiation of strains within the genus *Riemerella* [23]. Mass spectrometry is becoming an increasingly common tool, used not only in research centres but also in human and animal diagnostic laboratories, as it can cheaply, rapidly, and reliably identify microorganisms to the level of species or at least genus. For this reason, it is becoming an alternative to more time-consuming and expensive identification methods based on molecular biology, which require not only isolation of the microbe but also DNA extraction, amplification, and electrophoretic separation. Nevertheless, the *rpo*B gene is a very useful tool for identifying bacteria and phylogenetic analyses as it supplements results obtained by amplification of the 16S rRNA gene.

Comparison of *rpo*B gene sequences confirmed that *R. anatipestifer* is genetically separate from other representatives of the genus *Riemerella*, i.e., *R. columbina* and *R. columbipharyngis*, as well as from closely related strains belonging to the family *Weeksellaceae.*

*R. anatipestifer* is characterized more by the absence than the presence of specific phenotypic properties. Although *R. anatipestifer* is counted among bacteria which do not induce haemolysis on media with blood, nine isolates in our study induced type β haemolysis on CA (Columbia agar) medium following 48 h of incubation in microaerophilic conditions. This confirmed the results obtained by Hinz et al. [3], who observed β-haemolysis on agar in over 20% of isolates after 24–48 h of incubation. The ability of *R. anatipestifer* strains to induce haemolysis has also been observed by other researchers [9,25,26]. The biochemical properties of *R. anatipestifer* are often variable. Positive reactions are observed only for cytochrome oxidase and catalase, while the reactions to urase and gelatinase are varied and depend on the bacterial strain [27]. Extended incubation of commercial biochemical tests for 48–72 h resulted in positive results for gelatine hydrolysis as a positive reaction was confirmed after 48 h for 25 of the strains tested. There are reports that a positive result for indole production is possible [3], but none of the isolates used in the study exhibited this property. Similarly, none of them hydrolysed esculin. This reaction is one of the traits differentiating bacterial species within the genus *Riemerella*; unlike isolates of *R. anatipestifer*, *R. columbina*, in addition to producing pigment on media, also shows the ability to hydrolyse esculin. However, tests of samples from clinically healthy pigeons have shown that there are atypical strains of *R. columbina*, whose species was confirmed by sequencing of the 16S rRNA gene, which was not able to hydrolyse esculin [27]. The recently described species *R. columbipharyngis*, obtained from clinically healthy pigeons, like *R. anatipestifer* also does not hydrolyse esculin [23].

Analysis of the resistance profile of the isolates by testing the MICs of selected antibiotics and chemotherapeutics, including β-lactams, aminoglycosides, tetracycline, macrolides, fluoroquinolones, and polymyxins, revealed marked variation in the susceptibility of the *Riemerella* strains tested. Despite the introduction of new technologies to obtain data on the susceptibility of bacteria to antimicrobials, such as PCR, quantitative polymerase chain reaction (qPCR), next generation sequencing (NGS), and MALDI-TOF MS, recommended conventional methods for determining drug susceptibility are still commonly used [28]. In addition to diffusion tests, in which commercial antibiotic discs or strips are used, the most common methods include macro- and microdilution using solid or liquid media [29]. Dilution methods in broth or agar can be used to determine the MIC of antimicrobial agents. The MIC value is the basis for determining the category of the susceptibility of microbes to a given antibiotic, including bacteria for which inconclusive results have been obtained, especially when clinical threshold values for disc diffusion are not available. In contrast to the qualitative Kirby–Bauer method, the MIC value makes it possible to assess the degree of susceptibility to an antimicrobial substance [30]. The size of growth inhibition zones which would clearly indicate the degree of susceptibility of *R. anatipestifer* to a given antimicrobial substance using the Kirby–Bauer method has not been established, nor are there specific guidelines regarding the concentrations of antimicrobial substances indicating the susceptibility/resistance of *R. anatipestifer* strains. Therefore, we limited our analysis to the determination of MIC_50_ and MIC_90_ values (i.e., the concentration of the antibiotic/chemotherapeutic that would inhibit the growth of 50% and 90% of bacterial strains). In the absence of clinical threshold values, MIC values should provide information to the doctor responsible for the choice of antimicrobial agent for treatment. As expected, the highest percentage of resistant strains was observed for the aminoglycosides gentamicin and amikacin, for which the high MIC_50_ and MIC_90_ values may indicate cross-resistance to this group of antibiotics. This is also reflected in studies by other researchers. Studies by Sun et al. [31] and Chang et al. [12] on *R. anatipestifer* isolates from ducks in China showed high MIC values for gentamicin and amikacin but also for other aminoglycosides, i.e., neomycin, apramycin, kanamycin, and streptomycin (not included in the present study), with the MIC_50_ ranging from 32 to ≥128 μg/mL and MIC_90_ from 64 to ≥256 μg/mL. Only in a few strains of *R. anatipestifer* were resistance genes to aminoglycosides found to be present, including streptomycin. The *aac(6′)-Ib* gene, which determines resistance to amikacin, kanamycin, and tobramycin, is the most common resistance gene found in clinical isolates that are resistant to aminoglycosides. [32,33]. The presence of the *aac(3′)-IIc* gene, causing resistance to gentamicin and tobramycin in *R. anatipestifer,* has been also confirmed [31,32]. Despite the high percentage of gentamicin- and amikacin-resistant strains, presence of the resistance genes *aac(6′)-Ib* and *aac(3′)-IIc* was not detected in this study. To rule out any error in determining the amikacin/gentamicin MIC value, we additionally used the disc diffusion method. It is therefore possible that resistance to gentamicin and amikacin in our *R. anatipestifer* isolates is the result of the production of other aminoglycoside-modifying genes (e.g., ant-2) or other resistance mechanisms.

There are few reports of the use of the MIC method to determine the resistance of *R. anatipestifer* to colistin, an antibiotic from the group of polymyxins. Our results clearly demonstrate high resistance to this antibiotic, indicated by the high MIC_50_ and MIC_90_ values (≤128 μg/mL) for the isolates used in the study. This is confirmed by results obtained by Chang et al. [7] and Tzora et al. [13], who used the Kirby–Bauer method to show colistin resistance for all *R. anatipestifer* strains (n = 76) isolated from geese and ducks in Taiwan and strains isolated from a clinical case in chickens in Greece. In the last few years, there has been a significant change in the role of colistin in human and animal medicine; from a substance used exclusively in veterinary medicine, it has become a molecule of critical importance in human medicine. The widespread phenomenon of the increased resistance of bacterial strains to antibiotics and chemotherapeutics makes colistin a last-resort drug against human bacterial infections induced by multi-drug resistant bacteria, including *Pseudomonas aeruginosa*, *Acinetobacter baumannii,* and bacteria of the family *Enterobacteriaceae* (*Escherichia coli* and *Klebsiella pneumoniae*). The results described in this study and those reported by other authors cited in this paper clearly indicate that colistin, which in the European Union is available in veterinary drugs, either as the only active substance or as a component of combination drugs, should not be used in the treatment of septicaemia anserum exsudativa in poultry flocks due to the lack of positive therapeutic results and the risk of increased resistance. A fairly high MIC_90_ value of 32 μg/mL was obtained for the macrolide antibiotic erythromycin. A high percentage—nearly 84%—of *R. anatipestifer* strains isolated from geese and ducks showed resistance (31.6%) or intermediate susceptibility (52.2%) to erythromycin [7]. Xing et al. [34] reported that more than 53% of strains isolated from ducks in China were resistant to erythromycin. Our results and those of the other authors cited above indicate the growing phenomenon of acquired resistance to this chemotherapeutic, which initially was effective against *R. anatipestifer* strains [4,35,36]. The *ermF* gene, responsible for erythromycin resistance, was found in 14 *R. anatipestifer* isolates, with MIC values ranging from 4 to 64 µg/mL. The *ermF* gene, which codes for ribosomal methylase, can be the most frequently encoded gene that determines erythromycin resistance in *R. anatipestifer*, which is confirmed by the results of other authors [34].

Among the tetracycline resistance genes, *tet(A)*, *tet(B)*, and *tet(X)* were detected in isolates showing that the MIC values to tetracycline ranged from 1 to 8 µg/mL. The *tet(X)* gene is an enzymatic gene which encodes for an NADP-dependent monooxygenase that requires oxygen to degrade tetracycline and is the dominant mechanism conferring tetracycline resistance in *R. anatipestifer* isolates, which was confirmed by the results of Zhu et al. [37].

β-lactam antibiotics, which include amoxicillin, piperacillin, cefuroxime, and cefoperazone, showed effective antimicrobial activity against the *R. anatipestifer* isolates tested in the present study, with low MIC_90_ values not exceeding 2 μg/mL. Contrasting results were presented for several strains of *R. anatipestifer* isolated from young ducks for cefoperazone, with five of seven strains showing resistance [8]. In another study, susceptibility to amoxicillin and ceftiofur, a second-generation cephalosporin, was shown in *R. anatipestifer* strains from chickens [6]. The effectiveness of β-lactams was confirmed by results presented by Li et al. [38], who obtained a high susceptibility of *R. anatipestifer* isolates to ceftiofur and cefquinome, which are third- and fourth-generation cephalosporins, with MIC_50_/MIC_90_ values of 0.031–0.063/0.5 μg/mL. Although cephalosporins, as representatives of β-lactams, were used in the study and achieved the lowest concentration in inhibiting the growth of bacteria, they are not used to treat poultry; however, drugs of choice in the treatment of *R. anatipestifer* infections may include formulations containing amoxicillin. Despite low MIC values for β-lactams, including amoxicillin, the presence of the *bla_TEM_* resistance gene was detected in two strains, 62/23 and 63/23, whose MIC values for amoxicillin were low, at 1 and 0.5 μg/mL, respectively (Table 2 and Table S3). Similar results have been described by Sun et al. [31]. Low MIC values were also obtained for the fluoroquinolones, enrofloxacin and ciprofloxacin. The distribution of MICs for the population of bacteria used in the study was similar for both active substances, possibly due to cross-resistance within this group of drugs. Low MIC values were also obtained for chloramphenicol and for trimethoprim/sulfamethoxazole, which inhibits the synthesis and transformation of folic acid. The low MIC values for sulfamethoxazole with trimethoprim (MIC_50_ 0.125/2.38 μg/mL and MIC_90_ 1/19 μg/m) differ from the MIC values determined separately for these two antimicrobial agents; the MIC_50_ and MIC_90_ for sulfamethoxazole were 64 and 128 μg/mL, respectively, and the MIC_50_ and MIC_90_ for trimethoprim were 128 and >256 μg/mL [12]. This significant discrepancy may be due to the fact that trimethoprim in combination with sulfamethoxazole exerts synergistic bactericidal effects, which is equivalent to a better antimicrobial result [39]. Thus, enrofloxacin, which is a fluoroquinolone, amoxicillin, a β-lactam antibiotic, and also trimethoprim/sulfamethoxazole can be used in the treatment of septicaemia anserum exsudativa with a positive therapeutic effect in birds in the case of initial therapy, which is always empirical, introduced prior to typical targeted treatment once the result of a drug susceptibility test has been obtained.

The study clearly demonstrates the presence of *R. anatipestifer* strains among land and water fowl, which indicates a potential risk of infections in poultry flocks. The identification and taxonomic classification carried out in the study by analysing the *rpo*B gene sequence can be successfully used to identify *R. anatipestifer*. Although there have been few studies assessing the susceptibility of *R. anatipestifer* to antimicrobials by determining their MICs in liquid media, this technique is repeatable and less time-consuming than the determination of MICs using solid media and can be an auxiliary tool in the treatment of confirmed *R. anatipestifer* infections for targeted treatment of poultry. It is concerning that the comprehensive characterization of *R. anatipestifer* strains in the study showed high resistance to colistin, a crucial antibiotic in human medicine. It should be stressed that this is the first phenotypic characterization of *R. anatipestifer* strains from poultry flocks in Poland.

## 4. Materials and Methods

### 4.1. Isolation of Strains

The material for analysis was swabs from the lungs, trachea, heart, liver, infraorbital sinuses, buccal cavity, and hock joint of land fowl (chickens and turkeys) and waterfowl (ducks and geese) during anatomopathological examination carried out for diagnostic purposes at the veterinary clinic of the Department of Veterinary Prevention and Avian Diseases, Faculty of Veterinary Medicine, University of Life Sciences in Lublin, which did not require the approval of an ethics committee. The material for analysis was collected in the years 2015–2021, mainly from flocks in southeastern Poland. In addition, strains isolated at the LAB-VET Veterinary Diagnostics Laboratory in Tarnów Podgórny were added to the collection. Fifty-seven bacterial strains morphologically resembling *R. anatipestifer* were used in the analysis.

Strains were isolated on Columbia agar (CA, Oxoid Ltd., Basingstoke, UK) with the addition of 5% defibrinated sheep blood, calf serum (Biomaxima, Lublin, Poland), and also gentamicin (25 µg/mL). Bacterial isolates were simultaneously inoculated on selective MacConkey agar (Oxoid Ltd., Basingstoke, UK). The plates were incubated in microaerophilic conditions with 5% CO_2_ at 37 °C for 24–48 h. Preliminary phenotypic identification was based on the morphological structure of isolates grown on the medium. Biochemical traits were determined using a commercial test for identification of non-fermenting Gram-negative rods (API 20NE, Biomérieux, Craponne, France) according to the manufacturer’s instructions. All isolates were stored in commercial kits for storage of microorganisms and in Tryptone Soy Broth liquid medium (TSB; Oxoid Ltd., Basingstoke, UK) with 20% glycerol at −80 °C.

### 4.2. Species Identification by MALDI-TOF MS

The isolates were identified by MALDI-TOF MS (Bruker Daltonics, Bremen, Germany). The analysis was performed for bacterial isolates grown on CA agar for 24 h, which were subjected to protein extraction using absolute ethanol, formic acid, and acetonitrile (Sigma-Aldrich, Steinheim, Germany) according to the method described by Dudzic et al. [18]. Mass spectra were processed with the MALDI Biotyper 3.1 software package (Bruker Daltonics, Bremen, Germany) containing 8468 reference spectra, of which five corresponded to *R. anatipestifer* and one to *R. columbina.*

### 4.3. Identification of Bacteria by PCR

The DNA of the bacterial isolates was extracted using a commercial kit for DNA purification (GeneMatrix Bacterial & Yeast Genomic DNA Purfication Kit, EURx, Gdańsk, Poland) according to the manufacturer’s instructions. Identification was carried out using the uniplex PCR technique with primers for the 16S rRNA gene, i.e., 16S-UNI-L (5′-AGA GTT TGA TCA TGG CTC AG-3′) and Rcol-2 (5′-TGT TAC GAC TTA GCC CTA G-3′) [40], and species-specific primers: RA L-17 (5′-TAG CAT CTC TTG GAT TCC CTT C-3′) and RA R-354 (5′-CCA GTT TTT AAC CAC CAT TAC CC-3′) [24]. The reaction mixture used for PCR contained 1 µL (~20 ng) of the DNA, 12.5 µL of Dream Taq Green PCR Master Mix (Thermo Fisher Scientific, Cleveland, OH, USA), 1 µL of each primer (10 µM), and 9.5 µL RNAse-free water (EURx, Gdańsk, Poland). Amplification reactions were performed in the TProfessional Basic Thermocycler (Biometra GmbH, Göttingen, Germany) using the following programme: 1 cycle at 94 °C for 5 min; 32 cycles at 94 °C for 40 s, 56 °C for 40 s (for RA L-17/RA R-354); 52 °C for 40 s (for 16S-UNI-L/Rcol-2), 72 °C for 60 s, and a final cycle at 72 °C for 7 min. The PCR product length for 16S-UNI-L/Rcol-2 was 1460 bp, and the product length for RA L-17/RA R-354 was 338 bp. The PCR products obtained in 5 μL were separated by electrophoresis (100 V) on a 1% agarose gel in 1 × TBE buffer (Tris-borate-EDTA buffer) and visualized by SimplySafe staining (EURx, Gdańsk, Poland). Three reference strains, *R. anatipestifer* ATCC 11845, *R. columbina* DSM 16469, *R. columbipharyngis* DSM 24015, were included as controls.

### 4.4. Phylogenetic Analysis Based on Sequencing of rpoB Gene

The PCR product was used for phylogenetic analysis of the isolates, amplifying nucleotide sequences of the *rpo*B gene encoding DNA-dependent RNA polymerase β subunit, i.e., *rop*F (5′-TTAGATCCCATCAAGGCACG-3′) and *rop*R (5′-GAGCAGTTGGCAGGTCAGTT-3′), where the length of the reaction product was 686 bp [40]. The DNA sequence was determined by a commercial DNA sequencing service provider which used the Sanger method (Genomed, Warsaw, Poland). Sequences were assembled with CLC Genomics Workbench 7.0 (CLC bio, a Qiagen Company, Redwood City, CA, USA) and compared to reference sequences available in the GenBank database using the NCBI BLAST algorithm (http://www.ncbi.nlm.nih.gov/BLAST accessed on 17 July 2023). The phylogenetic tree, based on sequence analysis of the *rpo*B gene, was constructed on the basis of 68 sequences: the field isolates from poultry (n = 57), reference strains of *R. anatipestifer* (n = 6), *R. columbina* (n = 1), and *R. columbinapharyngis* (n = 1), and strains of related species of the family *Weeksellaceae*, *Cloacibacterium caeni*, *Chryseobacterium oryzae*, *and Elizabethkinigia meningoseptica*, whose *rpo*B sequences were obtained from the GenBank database (Table S3). Phylogenetic relatedness was inferred using the maximum likelihood method and the Tamura–Nei model [41]. The percentage of bootstrapping in which the associated taxa were clustered together is shown next to the branches. Initial tree(s) for the heuristic search were obtained automatically by applying the neighbour-joining (NJ) and BioNJ algorithms to a matrix of pairwise distances estimated using the maximum composite likelihood (MCL) approach and then selecting the topology with the superior log likelihood value. The tree is drawn to scale, with branch lengths proportional to the number of substitutions per site. The analysis was performed on a segment of 657 nucleotides; all positions with missing data (nucleotides) were eliminated (complete deletion option). Evolutionary analyses were conducted in MEGA X 11.0.13 software [42]. A list of strains whose *rpo*B gene sequences were used for comparative analysis and clustering is in the Supplementary Materials (Table S3).

### 4.5. Assessment of Antibiotic Profiles by the MIC Method

The resistance profiles of the isolates were evaluated by determining the minimum inhibitory concentration (MIC) for selected groups of antibiotics by the two-fold serial microdilution method according to the Clinical and Laboratory Standards Institute (CLSI) [29,43] using 96-well microplates. *R. anatipestifer* strains required enriched growth-supporting media and certain *Riemerella* strains required extended incubation time. For this purpose, the medium of choice was Mueller Hinton broth (MHB, (Oxoid Ltd., Basingstoke, UK) containing additionally 5% calf serum. The antibiotics tested were ciprofloxacin, enrofloxacin, amoxicillin, gentamicin, amikacin, tetracycline, chloramphenicol, colistin, erythromycin, cefuroxime sodium, cefoperazone, piperacillin, and trimethoprim/sulfamethoxazole. All antibiotics were purchased in the form of dry powder from Sigma-Aldrich, (Steinheim, Germany) except for chloramphenicol, which was obtained from Roth, (Karlsruhe, Germany).

A 50 µL volume of MHB with 5% calf serum was placed on the titration plate. A solution of antibiotic at a given concentration in the amount of 50 µL was added to the first well, and after thorough mixing 50 µL was transferred to the next wells until 10 different dilutions were obtained. The next-to-last well on the plate contained MHB with 5% calf serum and a bacterial suspension as a positive control. The last well on the plate contained only MHB with 5% calf serum as a negative control. Colonies of *R. anatipestifer* grown on TSB were suspended in 5 mL of 0.85% NaCl solution (final optical density at 600 nm was 0.5 McFarland standard, which corresponds to 5 × 10^5^ CFU/mL). Then, the bacterial suspension was suspended in a 1:100 ratio in MHB with 5% calf serum and 50 µL was added to each well (except the last). The plates were incubated for 24 h in microaerophilic conditions at 37 °C. Reference strains of *Escherichia coli* (ATCC 25922) and *Riemerella anatipestifer* (ATCC 11845) were used as controls, with the MICs for *Escherichia coli* (ATCC 25922) tested using MHB without serum, and the plates were incubated in aerobic conditions. The MIC test was performed in duplicate.

### 4.6. Detection of Resistance Genes

The presence of genes conferring resistance to aminoglycosides—*aac(6′)-Ib*, *aac(3′)-IIc*, *aac(3′)-IV*, *aph(3′)-VII*, *aadA*, and *strA/strB*; tetracyclines—*tet(A)*, *tet(B)* and *tet(X)*; β-lactams—*bla_TEM_*, *bla_OXA_*, *bla_CTX-M_*, *bla_SHV_*; sulfonamides—*sul1*, *sul2* and *sul3*; erytromycin*ermF*; trimethoprim—*dhfr1*; and chloramphenicol—*cat2*, *cmlA*, *flor*, were determined by PCR using the primers presented in Table S4. Amplification reactions were performed in the TProfessional Basic Thermocycler (Biometra GmbH, Göttingen, Germany) using the program described in Section 4.3, according to the annealing temperature for the individual primers (Table S4). The PCR products in a volume of 5 µL were separated by electrophoresis on a 1% agarose gel.

## Figures and Tables

**Table 1:** Distribution of minimum inhibitory concentrations of antibiotics for *R. anatipestifer.*

Antibiotic ^a^	Test Range (μg/mL)	Number of Isolates with MIC (μg/mL) of													MIC_50_ ^c^	MI_C90_ ^d^
		≤0.031	0.063	0.125	0.25	0.5	1	2	4	8	16	32	64	≥128		
AMX	0.125–128				24	19	3	6	2		1	2			0.5	2
TE	0.125–128				10	7	12	15	8	5					1	4
CN	0.125–128						2	3	3	1	4	6	8	30	128	128
AMK	0.125–128						1	1	2	5	4	3	4	37	128	128
CS	0.125–128							1	1	3	4	5	3	40	128	128
CL	0.125–128					6	5	27	7	7	4		1		2	8
ENR	0.125–128			11	10	14	2	2	7	3	5	1	2		0.5	16
CIP	0.125–128			11	6	8	11	4	6	8	1	2			1	8
ERY	0.125–128			4	13	16	4	3	3	4	1	5	4		0.5	32
CXM	0.031–16	19	16	20	1	1									0.063	0.125
CFP	0.031–16	17	4	12	19	5									0.125	0.25
PIP	0.031–16	2	8	21	8	13	3	2							0.125	0.5
Antibiotic	Test range (μg/mL)	Number of Isolates with MIC (μg/mL) of														
		≤0.06/1.19	0.125/2.38	0.25/4.75	0.5/9.5	1/19	2/38	4/76	8/152	16/228	≥32/304					
TR/S	0.06/1.19–32/304 ^b^	23	9	8	6	7		4							0.125/2.38	1/19

^a^ amoxicillin (AMX), tetracycline (TE), gentamicin (CN), amikacin (AMK), colistin (CS), chloramphenicol (CL), enrofloxacin (ENR), ciprofloxacin (CIP), erythromycin (ERY), cefuroxime (CXM), cefoperazone (CFP), piperacillin (PIP), trimethoprim/sulfamethoxazole (TR/S); ^b^ mixed in a 1:19 ratio; ^c,d^ MIC_50_/MIC_90_—concentration at which 50% or 90% of isolates, respectively, are inhibited.

**Table 2:** Presence of resistance genes of *Riemerella anatipestifer* isolates used in this study.

Strain ID	Resistance Genes	Strain ID	Resistance Genes
1/23	*tet(X)*	37/23	*-*
2/23	*tet(X)*	39/23	*tet(X)*
3/23	*ermF*	40/23	*tet(X)*
4/23	*tet(X)*	41/23	*tet(X)*
5/23	*tet(X)*, *tet(B)*	42/23	*-*
6/23	*tet(X)*	43/23	*-*
7/23	*-*	44/23	*tet(X)*, *sulI*
8/23	*tet(X)*, *ermF*	45/23	*tet(X)*
9/23	*-*	46/23	*tet(X)*
10/23	*tet(X)*	47/23	*tet(X)*
11/23	*tet(X)*, *ermF*	48/23	*-*
12/23	*tet(X)*	49/23	*tet(X)*
13/23	*tet(X)*	50/23	*tet(X)*
14/23	*-*	51/23	*aph(3′)-VII*
15/23	*tet(X)*	52/23	*-*
16/23	*tet(X)*	53/23	*tet(X)*, *tet(A)*,*ermF*, *cmlA*, *aph(3′)-VII*, *aac(3′)-IV*
17/23	*tet(X)*	54/23	*tet(X)*, *tet(A)*,*ermF*, *aph(3′)-VII*
20/23	*-*	55/23	*tet(X)*, *tet(A)*,*ermF*, *aph(3′)-VII*
22/23	*-*	56/23	*tet(X)*, *ermF*, *aph(3′)-VII*
23/23	*tet(X)*, *tet(B)*	58/23	*tet(X)*, *ermF*
25/23	*tet(X)*	59/23	*tet(X)*, *ermF*
26/23	*-*	61/23	*tet(X)*, *ermF*
27/23	*-*	62/23	*tet(X)*, *tet(A)*,*ermF*, *cmlA*, *blaTEM*, *aadA*, *strA/strB*
28/23	*tet(X)*	63/23	*tet(X)*, *tet(A)*,*ermF*, *aph(3′)-VII*, *aac(3′)-IV*, *blaTEM*, *aadA*, *strA/strB*
29/23	*tet(X)*	64/23	*tet(X)*, *tet(A)*, *cmlA*,
31/23	*tet(X)*, *tet(B)*, *cmlA*	65/23	*aph(3′)-VII*
33/23	*-*	69/23	*tet(X)*, *ermF*
34/23	*-*	70/23	*tet(X)*, *ermF*
35/23	*tet(X)*		

## Data Availability

The data presented in this study are contained within the article and Supplementary Materials.

## Supplementary Materials

The following supporting information can be downloaded at: https://www.mdpi.com/article/10.3390/antibiotics12121648/s1, Table S1: MALDI-TOF MS log(score) values for *Riemerella anatipestifer*; Table S2: Summary of individual MIC values for *Riemerella anatipestifer* isolates. Table S3: List of strains whose *rpo*B gene sequences were used for comparative analysis and clustering. References [44,45,46,47,48,49,50,51,52]; Table S4: Primers used in this study for PCR detection of the resistance genes. References [31,32,53,54,55,56,57,58,59].
