# Supplementary material for: Characterization of Riemerella anatipestifer Strains Isolated from Various Poultry Species in Poland
Source: Antibiotics (Basel). 2023 Nov 22;12(12):1648. doi: 10.3390/antibiotics12121648 (PMC10740677; doi:10.3390/antibiotics12121648)
Supplement: Supplementary file 1 [file antibiotics-12-01648-s001.zip › Table S1. MALDI-TOF MS log(score) values for R. anatipestifer.pdf]

Table S1. MALDI-TOF MS log(score) values for *R. anatipestifer*

Submission Number	Matched Pattern in the Bruker Daltonics Database	Log(score) Value
1/23	*Riemerella anatipestifer* GD47 GDD	2.131
2/23	*Riemerella anatipestifer* GD47 GDD	2.184
3/23	*Riemerella anatipestifer* DSM 15868T DSM	2.488
4/23	*Riemerella anatipestifer* DSM 15868T DSM	2.648
5/23	*Riemerella anatipestifer* DSM 15868T DSM	2.582
6/23	*Riemerella anatipestifer* DSM 15868T DSM	2.601
7/23	*Riemerella anatipestifer* DSM 15868T DSM	2.563
8/23	*Riemerella anatipestifer* GD47 GDD	2.119
9/23	*Riemerella anatipestifer* DSM 15868T DSM	2.416
10/23	*Riemerella anatipestifer* GD47 GDD	2.193
11/23	*Riemerella anatipestifer* DSM 15868T DSM	2.476
12/23	*Riemerella anatipestifer* DSM 15868T DSM	2.449
13/23	*Riemerella anatipestifer* DSM 15868T DSM	2.317
14/23	*Riemerella anatipestifer* DSM 15868T DSM	2.322
15/23	*Riemerella anatipestifer* DSM 15868T DSM	2.634
16/23	*Riemerella anatipestifer* DSM 15868T DSM	2.015
17/23	*Riemerella anatipestifer* DSM 15868T DSM	2.637
20/23	*Riemerella anatipestifer* DSM 15868T DSM	2.385
22/23	*Riemerella anatipestifer* DSM 15868T DSM	2.308
23/23	*Riemerella anatipestifer* DSM 15868T DSM	2.607
25/23	*Riemerella anatipestifer* DSM 15868T DSM	2.570
26/23	*Riemerella anatipestifer* GD49 GDD	2.384
27/23	*Riemerella anatipestifer* DSM 15868T DSM	2.005
28/23	*Riemerella anatipestifer* DSM 15868T DSM	1.924
29/23	*Riemerella anatipestifer* DSM 15868T DSM	2.515
31/23	*Riemerella anatipestifer* GD49 GDD	2.272
33/23	*Riemerella anatipestifer* DSM 15868T DSM	2.496
34/23	*Riemerella anatipestifer* DSM 15868T DSM	2.467
35/23	*Riemerella anatipestifer* DSM 15868T DSM	2.673
37/23	*Riemerella anatipestifer* DSM 15868T DSM	2.593
39/23	*Riemerella anatipestifer* DSM 15868T DSM	2.586
40/23	*Riemerella anatipestifer* DSM 15868T DSM	1.722
41/23	*Riemerella anatipestifer* DSM 15868T DSM	2.416
42/23	*Riemerella anatipestifer* DSM 15868T DSM	2.072
43/23	*Riemerella anatipestifer* DSM 15868T DSM	2.229

44/23	*Riemerella anatipestifer* DSM 15868T DSM	2.357
45/23	*Riemerella anatipestifer* DSM 15868T DSM	2.344
46/23	*Riemerella anatipestifer* DSM 15868T DSM	2.296
47/23	*Riemerella anatipestifer* DSM 15868T DSM	2.320
48/23	*Riemerella anatipestifer* GD47 GDD	2.015
49/23	*Riemerella anatipestifer* DSM 15868T DSM	2.232
50/23	*Riemerella anatipestifer* 11_00491_06 VAXM	2.088
51/23	*Riemerella anatipestifer* GD47 GDD	1.985
52/23	*Riemerella anatipestifer* DSM 15868T DSM	2.041
53/23	*Riemerella anatipestifer* GD47 GDD	2.044
54/23	*Riemerella anatipestifer* DSM 15868T DSM	2.109
55/23	*Riemerella anatipestifer* DSM 15868T DSM	2.160
56/23	*Riemerella anatipestifer* DSM 15868T DSM	2.174
58/23	*Riemerella anatipestifer* GD48 GDD	1.965
59/23	*Riemerella anatipestifer* DSM 15868T DSM	1.999
61/23	*Riemerella anatipestifer* DSM 15868T DSM	2.220
62/23	*Riemerella anatipestifer* DSM 15868T DSM	2.079
63/23	*Riemerella anatipestifer* DSM 15868T DSM	2.025
64/23	*Riemerella anatipestifer* DSM 15868T DSM	2.145
65/23	*Riemerella anatipestifer* DSM 15868T DSM	2.127
69/23	*Riemerella anatipestifer* GD47 GDD	2.126
70/23	*Riemerella anatipestifer* DSM 15868T DSM	2.174

According to Maldi Biotyper software version 3.1, a log score value between 2.300 and 3.000 indicates highly probable identification at the species level; between 2.000 and 2.290 means highly probable identification at the genus level and probable identification at the species level; between 1.700 and 1.990 allows probable identification to the genus level; below 1.700 does not allow for reliable identification.
