# Supplementary material for: Characterization of Riemerella anatipestifer Strains Isolated from Various Poultry Species in Poland
Source: Antibiotics (Basel). 2023 Nov 22;12(12):1648. doi: 10.3390/antibiotics12121648 (PMC10740677; doi:10.3390/antibiotics12121648)
Supplement: Supplementary file 1 [file antibiotics-12-01648-s001.zip › Table S3. List of strains whose rpoB gene sequences were used for comparative analysis and clustering.pdf]

Table S3. List of strains whose *rpo*B gene sequences were used for comparative analysis and clustering

Strain	Host/Source	Country of Isolation	Year of Isolation	GenBank Access no.	Source
1/23	nd	Poland	2015	OR574100	This study
2/23	duck	Poland	2015	OR574099	This study
3/23	duck	Poland	2015	OR574098	This study
4/23	nd	Poland	2016	OR574097	This study
5/23	nd	Poland	2016	OR574096	This study
6/23	nd	Poland	2016	OR574095	This study
7/23	duck	Poland	2015	OR574094	This study
8/23	duck	Poland	2015	OR574093	This study
9/23	nd	Poland	2015	OR574092	This study
10/23	duck	Poland	2015	OR574091	This study
11/23	duck	Poland	2015	OR574090	This study
12/23	duck	Poland	2015	OR574089	This study
13/23	nd	Poland	2015	OR574088	This study
14/23	nd	Poland	2017	OR574087	This study
15/23	goose	Poland	2017	OR574086	This study
16/23	goose	Poland	2017	OR574085	This study
17/23	goose	Poland	2017	OR574084	This study
20/23	goose	Poland	2016	OR574083	This study
22/23	nd	Poland	2017	OR574082	This study
23/23	chicken	Poland	2016	OR574081	This study
25/23	goose	Poland	2017	OR574080	This study
26/23	nd	Poland	2017	OR574079	This study
27/23	nd	Poland	2017	OR574078	This study
28/23	goose	Poland	2017	OR574077	This study
29/23	goose	Poland	2017	OR574076	This study
31/23	nd	Poland	2019	OR574075	This study
33/23	nd	Poland	2019	OR574074	This study

34/23	nd	Poland	2017	OR574073	This study
35/23	turkey	Poland	2017	OR574072	This study
37/23	nd	Poland	2017	OR574071	This study
39/23	turkey	Poland	2019	OR574070	This study
40/23	nd	Poland	2019	OR574069	This study
41/23	nd	Poland	2019	OR574068	This study
42/23	nd	Poland	2016	OR574067	This study
43/23	nd	Poland	2016	OR574066	This study
44/23	nd	Poland	2016	OR574065	This study
45/23	nd	Poland	2016	OR574064	This study
46/23	nd	Poland	2016	OR574063	This study
47/23	nd	Poland	2016	OR574062	This study
48/23	nd	Poland	2016	OR574061	This study
49/23	nd	Poland	2016	OR574060	This study
50/23	nd	Poland	2016	OR574059	This study
51/23	nd	Poland	2016	OR574058	This study
52/23	nd	Poland	2017	OR574057	This study
53/23	turkey	Poland	2020	OR574056	This study
54/23	turkey	Poland	2020	OR574055	This study
55/23	turkey	Poland	2020	OR574054	This study
56/23	turkey	Poland	2020	OR574053	This study
58/23	turkey	Poland	2020	OR574052	This study
59/23	turkey	Poland	2020	OR574051	This study
61/23	turkey	Poland	2020	OR574050	This study
62/23	turkey	Poland	2020	OR574049	This study
63/23	turkey	Poland	2020	OR574048	This study
64/23	turkey	Poland	2020	OR574047	This study
65/23	chicken	Poland	2021	OR574046	This study
69/23	turkey	Poland	2021	OR574045	This study

70/23	turkey	Poland	2021	OR574044	This study
R. columbina DSM 16469	reference strain *R. columbina* DSM 16469	Poland	2023	OR614026	This study
R. columbipharyngis DSM24015	reference stain *R. columbipharyngis* DSM24015	Poland	2023	OR614027	This study
R. anatipestifer RCAD0125 GB	duck	China	2012	CP121209.1	[44]
R. anatipestifer NCTC 11014	duck	United Kingdom	1932	LT906475.1	[45]
R. anatipestifer ATCC 11845	duck	China	2012	CP003388.1	[46]
R. anatipestifer RA-LZ01	duck	China	2012	CP045564.1	[47]
R. anatipestifer SCVM0004	duck	China	2023	CP104076.1	[48]
R. anatipestifer RCAD0416	duck	China	2017	CP073239.1	[49]
Cloacibacterium caeni Isolate 1	wastewater	Norway	2021	OU015319.1	[50]
Chryseobacterium oryzae ADR-1 GB	rice punch	South Korea	2022	CP094529.1	[51]
Elizabethikingia meningoseptica G4120 GB	human	France	1983	CP016378.1	[52]

nd - no data
