# Supplementary material for: Characterization of Riemerella anatipestifer Strains Isolated from Various Poultry Species in Poland
Source: Antibiotics (Basel). 2023 Nov 22;12(12):1648. doi: 10.3390/antibiotics12121648 (PMC10740677; doi:10.3390/antibiotics12121648)
Supplement: Supplementary file 1 [file antibiotics-12-01648-s001.zip › Table S4. Primers used in this study for PCR detection of the resistance genes..pdf]

Table S4. Primers used in this study for PCR detection of the resistance genes.

Genes	Primers	Sequences(5′-3′)	Amplicon Size (bp)	Temp (°C)	Reference
aac(6′)-Ib	aac(6′)-Ib-F<br>aac(6′)-Ib-R	TTG CGA TGC TCT ATG AGT GG CTA<br>CTC GAA TGC CTG GCG TGT TT	482	56	[53]
aac(3′)-IIc	aac(3′)-IIc-F<br>aac(3′)-IIc-R	AAC CGG TGA CCT ATT GAT GG<br>TGT GCT GGC ACG ATC GGA GT	774	56	[31]
aph(3′)-VII	aph(3′)-VII-F<br>aph(3′)-VII-R	TCC ATA GGA TGG CAA GAT CC<br>TTC AAC GGG AAA CGT CTT GC	690	54	[31]
aac(3′)-IV	aac(3-′)-IV-F<br>aac(3′)-IV-R	GGC CAC TTG GAC TGA TCG AG<br>GCG GAT GCA GGA AGA TCA AC	609	56	[31]
aadA	aadA-F<br>aadA-R	GTG GAT GGC GGC CTG AAG CC<br>AAT GCC CAG TCG GCA GCG	525	60	[54]
strA/strB	strA-F<br>strB-R	ATG GTG GAC CCT AAA ACT CT<br>CGT CTA GGA TCG AGA CAA AG	893	55	[54]
tet(A)	tet(A)-F<br>tet(A)-R	GCG CCT TTC CTT TGG GTT CT<br>CCA CCC GTT CCA CGT TGT TA	831	55	[55]
tet(B)	tet(B)-F<br>tet(B)-R	CATTAATAGGCGCATCGCTG<br>TGAAGGTCATCGATAGCAGG	930	54	[56]
tet(X)	tet(X)-F<br>tet(X)-R	ATG ACA ATG CGA ATA GAT ACA GAC A<br>CAA TTG CTG AAA CGT AAA GTC	1167	55	[57]
bla$_{TEM}$	bla$_{TEM}$-F<br>bla$_{TEM}$-R	CGC CGC ATA CAC TAT TCT CAG AAT GA<br>ACG CTC ACC GGC TCC AGA TTT AT	445	60	[58]
bla$_{OXA}$	bla$_{OXA}$-F<br>bla$_{OXA}$-R	ACA CAA TAC ATA TCA ACT TCG C<br>AGT GTG TTT AGA ATG GTG ATC	813	60	[58]
bla$_{CTX-M}$	bla$_{CTX-M}$-F<br>bla$_{CTX-M}$-R	ATG TGC AGY ACC AGT AAR GTK ATG GC<br>TGG GTR AAR TAR GTS ACC AGA AYC AGC GG	593	60	[58]
bla$_{SHV}$	bla$_{SHV}$-F<br>bla$_{SHV}$-R	CTT TAT CGG CCC TCA CTC AA<br>AGG TGC TCA TCA TGG GAA AG	237	60	[58]
sul1	sul1-F<br>sul1-R	GTG ACG GTG TTC GGC ATT CT<br>TCC GAG AAG GTG ATT GCG CT	779	56	[56]
sul2	sul2-F<br>sul2-R	CCT GTT TCG TCC GAC ACA GA<br>GAA GCG CAG CCG CAA TTC AT	435	55	[55]
sul3	sul3-F<br>sul3-R	GAG CAA GAT TTT TGG AAT CG<br>CAT CTG CAG CTA ACC TAG GGC TTT GGA	880	53	[56]
ermF	ermF-F<br>ermF-R	ACC ACT TTC CAG TCT TAC GAA G<br>CGA CTT TGA ACT ACG AAG GAT G	994	55	[32]
dhfrI	dhfr-F<br>dhfr-R	AAG AAT GGA GTT ATC GGG AAT G<br>GGG TAA AAA CTG GCC TAA AAT TG	391	50	[59]
cat2	cat2-F<br>cat2-R	AAC GGC ATG ATG AAC CTG AA<br>ATC CCA ATG GCA TCG TAA AG	547	55	[56]
cmlA	cmlA-F<br>cmlA-R	CGC CAC GGT GTT GTT GTT AT<br>GCG ACC TGC GTA AAT GTC AC	394	55	[56]
flor	flor-F<br>flor-R	CTG AGG GTG TCG TCA TCT AC<br>GCT CCG ACA ATG CTG ACT AT	673	55	[56]
